# Nutrient, Fibre, and FODMAP Intakes and Food-related Quality of Life in Patients with Inflammatory Bowel Disease, and Their Relationship with Gastrointestinal Symptoms of Differing Aetiologies

**DOI:** 10.1093/ecco-jcc/jjab116

**Published:** 2021-07-03

**Authors:** Selina R Cox, Hazel Clarke, Majella O’Keeffe, Patrick Dubois, Peter M Irving, James O Lindsay, Kevin Whelan

**Affiliations:** 1 King’s College London, Faculty of Life Sciences and Medicine, Department of Nutritional Sciences, London, UK; 2 King’s College Hospital NHS Foundation Trust, Department of Gastroenterology, London, UK; 3 Guy’s and St Thomas’ NHS Foundation Trust, Department of Gastroenterology, London, UK; 4 Barts Health NHS Trust, Department of Gastroenterology, Royal London Hospital , London, UK

**Keywords:** Inflammatory bowel disease, nutrition, diet, FR-QoL, food-related quality of life

## Abstract

**Background and Aims:**

Certain foods are reported as gut symptom triggers in inflammatory bowel disease [IBD], and fructans are shown to worsen non-inflammatory symptoms in inactive IBD, which may result in self-imposed dietary restrictions. The aim of this study was to investigate nutrient and FODMAP intakes, and the relationship between gut symptoms and dietary intake, in IBD.

**Methods:**

Nutrient, fibre, and FODMAP intakes were estimated using 7-day food records in patients with active IBD [Active IBD], inactive IBD with non-inflammatory gut symptoms [Inactive IBD-GI], inactive IBD without gut symptoms [Inactive IBD], and healthy controls. Nutrient intakes, numbers of participants achieving national recommendations, and food-related quality of life [FR-QoL] were compared across study groups.

**Results:**

Food diaries were obtained from 232 patients with IBD [65 Active IBD, 86 Inactive IBD-GI, 81 Inactive IBD] and 84 healthy controls. Patients with Active IBD had significantly lower intakes of numerous micronutrients, including iron, folate, and vitamin C, compared with controls. All IBD groups consumed less total fibre [4.5 to 5.8 g/day] than controls [*p* = 0.001], and total FODMAP and fructan intakes were lower in Active IBD compared with controls. Strikingly, FR-QoL was significantly lower in all IBD groups compared with controls [all *p* = 0.001].

**Conclusions:**

This study revealed lower intakes of fibre, FODMAPs, and micronutrients, in addition to poorer FR-QoL, in Active IBD and Inactive IBD-GI with gut symptoms compared with healthy controls. Future research should address dietary restrictions responsible for these differences.

## 1. Introduction

Inflammatory bowel disease [IBD] has the potential to significantly impact on dietary intake. Patients with IBD report numerous nutritional problems, including difficulties with body weight, lethargy, foods identified to trigger gastrointestinal [GI] symptoms, social activities, and micronutrient deficiencies.^1^ These nutritional problems can have a profound psychosocial impact,^2^ and problems of food-related quality of life [FR-QoL] are prevalent in IBD.^3^ A priority-setting partnership consisting of patients and clinicians identified several dietary research priorities for IBD.^4^ During the priority setting, of all the questions pertaining to diet, 72% were raised by patients, including those regarding the role of diet in managing gut symptoms, in disease relapse, and in disease treatment.

Patients with IBD are at greater risk of protein-energy malnutrition and specific micronutrient deficiencies, in particular iron, vitamin B_12_ and vitamin D, than the general population.^5–8^ Malnutrition is particularly prevalent during active IBD. Patients with active IBD admitted to hospital were five times more likely to be malnourished compared with non-IBD admissions, and this difference was greater in penetrating Crohn’s disease [CD] and patients who had previously undergone bowel resection.^9^ The mechanisms of malnutrition in active IBD are thought to include increased nutrient requirements, reduced intestinal nutrient absorption, and increased intestinal nutrient losses, as well as impaired dietary intake.^10^

Numerous studies have investigated nutrient intakes in IBD, and although their findings vary greatly, in general these studies have revealed inadequate intakes of energy, fibre, vitamins C, D, B_1_ and B_6_, calcium, β-carotene, phosphorus, and magnesium, among others.^11–17^ Many of these studies are small in sample size, for example recruiting only 54–126 patients with IBD.^11–17^ Findings from dietary surveys in IBD also vary greatly as a result of methodological heterogeneity, including heterogeneity in the groups compared [e.g. IBD vs control; active IBD vs inactive IBD; CD vs ulcerative colitis], absence of healthy controls against whom to compare intakes, limited dietary assessment methods [e.g. food frequency questionnaires], and inconsistent nutrient reporting.^17,18^

Approximately a third of patients with IBD continue to experience gut symptoms in the absence of objective evidence of gastrointestinal [GI] inflammation. These represent non-inflammatory gut symptoms, that might otherwise be classified as irritable bowel syndrome [IBS].^19^ Dietary triggers of gut symptoms have been reported in 60% of patients with IBD.^20^ Therefore, patients with IBD in conjunction with non-inflammatory gut symptoms may have altered dietary intake; however, this has not been investigated specifically in this patient group.

Fermentable carbohydrates, or FODMAPs [fermentable oligosaccharides, disaccharides, monosaccharides, and polyols], are partially or fully indigestible in the GI tract and increase luminal water and gas through osmotic action and fermentation.^21^ In IBS, fermentable carbohydrates increase gut symptoms through luminal distension and potentially other mechanisms relating to changes in gut microbiota composition and output [e.g. alterations to short-chain fatty acid generation],^21^ and in some patients with IBD experiencing non-inflammatory gut symptoms that are adequately controlled with a low FODMAP diet, a fructan challenge can re-trigger symptoms.^22^ Data on FODMAP intakes in IBD are limited to two studies; one case-control study^23^ and one uncontrolled cross-sectional study,^24^ both showing lower intakes of some FODMAPs in IBD. Neither study estimated nor adjusted for participants’ energy intakes, making it impossible to attribute differences in FODMAP intakes to specific avoidance of high-FODMAP foods, rather than to a reduced overall food intake in IBD. Intakes of FODMAPs could be influenced by dietary restrictions commonly observed in IBD, including restriction of dairy products [containing lactose], beans [containing galacto-oligosaccharides; GOS] and certain fruits and vegetables [containing fructose and polyols].^25,26^ Since FODMAPs are prebiotic carbohydrates, dietary restriction may reduce potentially immune-regulatory bacteria in IBD,^27^ and therefore it is crucial to investigate the prevalence of intentional or unintentional restriction of FODMAPs in IBD both during active disease and in those with non-inflammatory gut symptoms.

Given the lack of consistency in methodology and findings of dietary surveys in IBD, the lack of assessment of the impact of inflammatory and non-inflammatory symptoms on intake, and the association of these with FR-QoL, a comprehensive assessment of nutrient intakes using a robust dietary assessment in a large group of patients with active and inactive CD and ulcerative colitis [UC] [both with and without non-inflammatory gut symptoms] is warranted. Therefore, the aim of this case-control study was to investigate nutrient and FODMAP intakes and FR-QoL in patients with active IBD [inflammatory gut symptoms], patients with inactive IBD with non-inflammatory gut symptoms [non-inflammatory gut symptoms] and patients with inactive IBD without gut symptoms [no gut symptoms] compared with healthy controls [no gut symptoms].

## 2. Materials and Methods

### 2.1. Study design and participants

This was a case-control study of 7-day dietary intake measurement in patients with: active IBD [Active IBD]; inactive IBD with non-inflammatory gut symptoms [Inactive IBD-GI]; inactive IBD without gut symptoms [Inactive IBD]; and healthy controls [HC]. Patients with CD and ulcerative colitis [UC] were recruited from three large gastroenterology clinics [Guy’s and St Thomas’ NHS Foundation Trust, Barts Health NHS Trust, King’s College Hospital NHS Foundation Trust] in London, UK. Healthy controls were staff and students from King’s College London and Guy’s and St Thomas’ NHS Foundation Trust, in order that they might reflect similar geographical and demographic profiles. To limit sampling bias associated with recruiting university and hospital students and academic and clinical staff, attempts were made to recruit from a diverse staff population including emailing and posting leaflets to professional services staff, administration staff, and building maintenance staff.

Patients with IBD [Active IBD, Inactive IBD-GI, and Inactive IBD] had common inclusion and exclusion criteria in addition to criteria specific for each group. Common inclusion criteria across all three IBD groups were that patients should be aged 18–75 years, with IBD [CD or UC] diagnosed through standard clinical, histological, and radiological criteria at least 3 months before screening. Common exclusion criteria across all three IBD groups were: stricturing CD; extensive intestinal resection; a current stoma; other gut disorders; significant comorbidities; currently following a special or restrictive diet unrelated to IBD; and pregnancy or lactation.

For the Active IBD group, a Harvey‐Bradshaw Index [HBI] ≥5 for CD or a Simple Clinical Colitis Activity Index [SCCAI] ≥4 for UC was required together with at least one objective measure of active disease within the preceding 4 weeks, defined as: C-reactive protein [CRP] ≥10 mg/L; or faecal calprotectin ≥250 μg/g; or endoscopic or imaging investigations indicating active disease.

Identifying ‘non-inflammatory’ gut symptoms in IBD is challenging, since these symptoms are often indistinguishable from those relating to GI inflammation, and furthermore, low-grade inflammation may not always be reflected in standard blood and stool tests. In this study, patients in the inactive IBD-GI group were required to have both an objective measure of inactive disease [and no objective evidence of active disease] within the preceding 4 weeks [CRP 10 mg/L; faecal calprotectin 250 μg/g; or endoscopic or imaging investigations indicating inactive disease], in addition to the presence of gut symptoms meeting the Rome III criteria for either IBS [diarrhoea predominant, mixed subtype, or unsubtyped IBS], functional bloating, or functional diarrhoea.

For the Inactive IBD group, an HBI ≤3 for CD or SCCAI ≤2 for UC was required in addition to at least one objective measure of inactive disease within the preceding 4 weeks [as described above]. Patients were excluded from the Inactive IBD group if they met Rome III criteria. Patients who had changes in IBD medications within 2 weeks of screening were excluded from both the Inactive IBD-GI and Inactive IBD groups, to ensure stable inactive disease.

The HBI and SCCAI were chosen as reliable and valid non-invasive measures of IBD activity^28^ and were used in conjunction with the presence/absence of objective markers. Patients with borderline scores [HBI of 4; SCCAI of 3] were excluded in order to create clear distinction between patients in the Active IBD group and those in the Inactive IBD group.

Healthy controls were aged 18–75 years and were excluded if they had IBD or another gut disorder, gut symptoms meeting the Rome III criteria for IBS, functional bloating or functional diarrhoea, a previous GI resection, significant comorbidities, were following a special or restrictive diet, or were pregnant or breastfeeding.

Research ethics committee approval was received from the London City & East ethics committee [reference 16/LO/0976].

The data underlying this article will be shared on reasonable request to the corresponding author.

### 2.2. Trial protocol

Patients with IBD were recruited through gastroenterology outpatient clinics and biologic infusion clinics. Potentially suitable patients were identified by gastroenterologists, IBD nurses and IBD pharmacists, and referred to the researcher for screening. Healthy control participants were recruited through circular email, posters and flyers at King’s College London or Guy’s and St Thomas’ NHS Foundation Trust, and potentially eligible healthy controls were invited to attend a screening and study visit at King’s College London.

Following informed consent and screening, demographic information was collected including age, gender, ethnicity, educational status, andsmoking history, together with basic anthropometry [weight, height, body mass index]. For patients with IBD, clinical information was also recorded including disease activity [HBI/SCCAI], Montreal classification,^29^ nad current medications, and in the Inactive IBD-GI group Rome III allocation was also recorded.

A 7-day food record was provided together with the food-related quality of life 29-item questionnaire [FR-QoL-29]^30^ and patient-perceived control of IBD [IBD-control] questionnaire,^31^ which were completed once during the week of food record completion. Patients either returned the food record and questionnaires to the researcher during a follow-up study visit, or posted it in a pre-stamped, addressed envelope.

### 2.3. Outcome measures and rationale

#### 2.3.1. Dietary assessment

A gold-standard 7-day food record was chosen, rather than the typical 3-day or 4-day food record previously used,^11,13,17,18^ to improve precision of global food intake assessment and tocontrol for inter-diurnal variation in eating behaviour, particularly important for less commonly consumed foods. Exceeding 7 days could compromise the quality of record completion and agreement to participate. Participants in all study groups were instructed on food diary recording by the lead researchers [registered dietitians]. They were asked to complete the food record prospectively for 7 consecutive days, and not during a holiday or a time unlikely to reflect normal dietary intake. Participants were asked to record the name, brand, cooking method, and portion size of all foods and drinks consumed. Portion size was recorded using measures written on packets or tins of packaged foods, household measures [e.g. tablespoons, cup], and food photographs for amorphous foods [e.g. curries, stews].^32^ All patients were contacted 2–3 days after enrolment to monitor and encourage compliance with food record completion. Immediately following return of the food record, the researchers checked the records for completeness and contacted participants for further details if required, in order to improve data accuracy.^33^

The data from the 7-day food records were entered into a cloud-based nutrient analysis software [Nutritics©, Dublin, Ireland] based upon McCance and Widdowson’s composition of foods integrated dataset.^34^ The software was regularly updated with composite foods, based upon information provided by manufacturers or publications. Dietary data were entered by one registered dietitian trained in the software. Potential coding errors were identified by calculating average daily intakes of energy, carbohydrate, protein, fat, fibre, calcium, iron, and vitamin C for each participant, and any falling outside the 2.5th or 97.5th percentile ranges for age and gender-matched national averages [based upon the 2008/09 and 2011/12 National Diet and Nutrition Survey] were checked against the food record source data for potential errors in data entry.

Intakes of total FODMAPs [calculated as the sum of all individual FODMAPs, including excess fructose but not total fructose], and individual FODMAPs [fructans, galacto-oligosaccharides, lactose, total fructose, excess fructose, sorbitol, and mannitol] were measured by entering the 7-day food diary into a proprietary database established at Monash University [the Monash FODMAP Calculator, Monash University, Australia].

Nutrient intake was expressed as units/day [d] and also as the proportion of participants achieving dietary reference values. For micronutrients, this was defined as meeting or exceeding reference nutrient intake [RNI] outlined by the United Kingdom Scientific Advisory Committee on Nutrition.^35–37^ For calcium, intakes were compared with both the RNI for the general adult population [700 mg/d] and the higher requirement for IBD recommended by the British Society of Gastroenterology [1000 mg/day].^38^ Intakes of fibre (non-starch polysaccharide [NSP]; total fibre [Association of Analytical Chemists] [AOAC]) and FODMAPs are presented as both g/d and as g/1000 kcal/d, the latter enabling comparison of intakes adjusted for total food intake.

#### 2.3.2. Food-related quality of life and IBD-control

Food-related quality of life encompasses the pleasure derived from food and the social activities involving eating and drinking.^39^ Inflammatory bowel disease can have a profound impact on the psychosocial aspects of food and mealtimes^2^ and impaired FR-QoL is prevalent in IBD, which is associated with lower intake of key nutrients. The FR-QoL-29 is a validated 29-item questionnaire pertaining to the impact of IBD on enjoyment of mealtimes and psychosocial activities involving food, eating and drinking. In healthy controls, a modified version of the FR-QoL-29 was used with the term ‘IBD’ changed to ‘digestion’.

The IBD-control questionnaire is a validated patient-reported outcome measure to capture patient-perceived control of IBD, encompassing questions regarding perceived usefulness of medications, current gut symptoms, and impact on quality of life.^31^ IBD-control was therefore not completed by the healthy control group.

### 2.4. Statistics

The target sample size was calculated based upon a previous study of fructan intakes in IBD.^23^ Based upon the mean fructan intakes observed in the active IBD (3.1 g/d, standard deviation [SD] 2.0 g/d) and healthy control [4.2 g/d] groups in that previous study, a sample size estimation established that 80 participants per group would be required to estimate a mean difference in fructan intakes of 1.1 g/d between the active IBD and healthy controls groups, with a power of 80% and a two-sided significance of 0.8% [to incorporate post hoc adjustment for multiple comparisons between the four study groups].^40^

Data analysis was performed using IBM SPSS Statistics for Windows Version 26.0, after the final participant’s data were collected. Before statistical analysis, continuous data were explored for normality via visual inspection of histograms. Continuous data across the four groups were compared using analysis of variance [ANOVA] or Kruskal‐Wallis tests, as appropriate depending upon normality of distribution, with pairwise comparisons and Bonferroni correction for multiple comparisons between groups. The chi square test was used to compare categorical variables across groups, with pairwise comparisons and Bonferroni correction for multiple comparisons between groups. Pearson or Spearman correlations were used to investigate correlations between outcomes; *p*-values ≤0.05 were considered statistically significant.

## 3. Results

**Figure 1.**
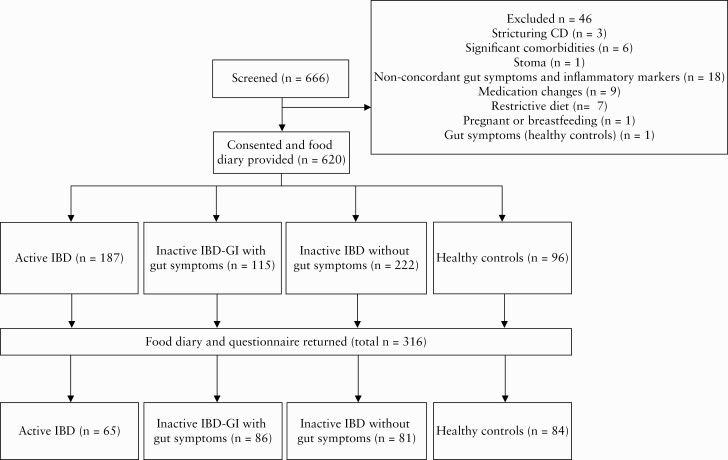
Diagram of patient flow through the study.

Recruitment took place between September 2016 and October 2019. In total 316 food diaries were returned [51% response rate], and these consisted of 65 with Active IBD, 86 with Inactive IBD-GI, 81 Inactive IBD and 84 healthy controls [Figure 1].

Demographic characteristics of the four study groups are displayed in Table 1. Across the groups, there was a significant difference in age [*p* = 0.034], with healthy controls (mean age 34 y [years], SD 13) being significantly younger than patients with active IBD [40 y, SD 12, *p* = 0.025]. There was a significant difference in educational level across groups [*p* = 0.005]. More healthy controls had their highest education qualification above compulsory school level than patients in the Inactive IBD-GI group [*p* = 0.006] and Inactive IBD group [*p* = 0.025]. Across groups there was a significant difference in smoking status [*p* = 0.007]; compared with healthy controls, there were fewer non-smokers in the Inactive IBD-GI group [*p* = 0.006], and more previous smokers in the Active IBD group [*p* = 0.026].

**Table 1.** Demographic characteristics across the four study groups.

	Active IBD [*n* = 65]	Inactive IBD-GI with gut symptoms [*n* = 86]	Inactive IBD without gut symptoms [*n* = 81]	Healthy controls [*n* = 84]	*p*-value
Age [years], mean [SD]	40 [12]^a^	37 [12]^a^^,^^b^	38 [14]^a^^,^^b^	34 [13]^b^	**0.034**
Female, *n* [%]	30 [46]	47 [55]	45 [56]	56 [67]	0.090
Ethnicity,^c^*n* [%]					0.111
White	52 [80]	73 [85]	71 [88]	56 [67]	
Mixed/multiple ethnic groups	3 [5]	2 [2]	1 [1]	7 [8]	
Asian/Asian British	6 [9]	7 [8]	5 [6]	10 [12]	
Black/African/Caribbean/Black British	3 [5]	3 [4]	1 [1]	5 [6]	
Other ethnic group [e.g. Arab]	1 [2]	1 [1]	3 [4]	6 [7]	
Maximum educational attainment, *n* [%]					**0.005**
No formal qualifications	4 [6]	2 [2]	4 [5]	1 [1]	
Vocational	2 [3]	6 [7]	2 [3]	2 [2]	
School-level [e.g. GCSE]	7 [11]^a^^,^^b^	15 [17]a	12 [15]^a^	2 [2]^b^	
Advanced [e.g. A level]	11 [17]	13 [15]	10 [12]	16 [19]	
University degree	22 [43]	40 [47]	33 [41]	30 [36]	
Postgraduate degree	9 [14]	10 [12]	17 [21]	23 [27]	
PhD	4 [6]^a^^,^^b^	0 [0]^a^	3 [4]^a^^,^^b^	10 [12]^b^	
Smoking status, *n* [%]					**0.007**
Current smoker	3 [5]	10 [12]	4 [5]	4 [5]	
Previous smoker	20 [31]^a^	24 [28]^a^^,^^b^	13 [16]^a^^,^^b^	10 [12]^b^	
Non-smoker	42 [65]^a^^,^^b^	52 [61]^a^	64 [79]^a^^,^^b^	70 [83]^b^	
Body weight [kg], mean [SD]	71.0 [13.6]	71.5 [14.1]	71.4 [15.0]	67.9 [17.1]	0.351
Height [m], mean [SD]	1.7 [0.1]	1.7 [0.1]	1.7 [0.1]	1.7 [0.1]	0.877
Body mass index [kg/m^2^], mean [SD]	24.2 [4.7]	24.0 [4.7]	24.5 [4.4]	23.0 [5.5]	0.248

*p*-values in bold are statistically significant (*p* < 0.05).

Continuous variables were compared across groups using one-way ANOVA and categorical variables were compared using the chi square test.

IBD, inflammatory bowel disease; GI, gastrointestinal; SD, standard deviation; GCSE, General Certificate of Secondary Education; A-level, advanced level; ANOVA, analysis of variance.

Groups with differing superscript letters are significantly different at the 0.05 level following pairwise comparisons with Bonferroni *post hoc* correction.

^c^Ethnic groups coded using ethnicity harmonised standard, Government Statistical Service, UK: [https://gss.civilservice.gov.uk/policy-store/ethnicity/#questions-england].

As expected, HBI, SCCAI, and IBD-control scores were significantly different across groups, with higher scores in Active IBD compared with both Inactive IBD-GI and Inactive IBD, and in Inactive IBD-GI compared with Inactive IBD [Table 2]. Across groups there were differences in the proportions of patients taking steroids [*p* = 0.001] and mesalazine [*p* = 0.002]. Significantly fewer patients with Inactive IBD-GI were taking mesalazine at recruitment compared with Active IBD [*p* = 0.008] and Inactive IBD [*p* = 0.010], and more patients with Active IBD were taking steroids compared with Inactive IBD [*p* = 0.002].

**Table 2.** Clinical characteristics among the IBD groups.

	Active IBD [*n* = 65]	Inactive IBD-GI with gut symptoms [*n* = 86]	Inactive IBD without gut symptoms [*n* = 81]	*p*-value
Medications, *n* [%]				
Mesalazine	31 [48]^a^	33 [38]^b^	40 [49]^a^	**0.002**
Thiopurines	21 [32]	35 [41]	36 [44]	0.149
Biologics	22 [34]	35 [41]	30 [37]	0.632
Steroids	13 [20]^a^	0 [0]^b^	3 [4]^b^	**<0.001**
Years since diagnosis, mean [SD]	10 [8]	10 [10]	13 [10]	0.076
IBD-control score, mean [SD]	52 [27]^a^	79 [23]^b^	106 [17]^c^	**<0.001**
Crohn’s disease, *n* [%]	25 [38]	48 [56]	44 [54]	0.074
Harvey‐Bradshaw Index [CD only], mean [SD]	7 [3]^a^	4 [2]^b^	1 [1]^c^	**<0.001**
Crohn’s disease location, *n* [%]				0.474
Ileal	7 [11]	16 [19]	8 [10]	
Colonic	6 [9]	12 [14]	16 [20]	
Ileocolonic	12 [18]	20 [23]	21 [26]	
Perianal disease	3 [5]^a^	9 [11]^a^	10 [12]^a^	**0.039**
Crohn’s disease behaviour, *n* [%]				0.512
Non-stricturing, non-penetrating	14 [22]	25 [29]	25 [31]	
Stricturing	10 [15]	15 [17]	12 [15]	
Penetrating	1 [2]	6 [7]	8 [10]	
Surgery [CD only], *n* [%]	12 [18]	17 [20]	23 [28]	0.273
Ulcerative colitis, *n* [%]	40 [62]	38 [44]	37 [46]	0.074
Simple Clinical Colitis Activity Index [UC only], mean [SD]	8 [2]^a^	3 [2] ^b^	1 [1] ^c^	**<0.001**
Ulcerative colitis extent, *n* [%]				0.083
Proctitis	6 [11]	12 [14]	5 [6]	
Distal	23 [42]	14 [16]	14 [17]	
Extensive	11 [20]	12 [14]	17 [21]	
Ulcerative colitis severity, *n* [%]				**<0.001**
Remission	5 [9]	7 [8]	36 [44]	
Mild	16 [29]	3 [3]	0 [0]	
Moderate	17 [31]	2 [2]	0 [0]	
Severe	1 [2]	0 [0]	0 [0]	
Rome III criteria fulfilled, *n* [%]				
IBS-D	-	22 [26]	-	
IBS-M	-	4 [5]	-	
IBS-U	-	4 [5]	-	
Functional bloating	-	49 [57]	-	
Functional diarrhoea	-	7 [8]	-	

*p*-values in bold are statistically significant (*p* < 0.05).

Continuous variables were compared across groups using one-way ANOVA and categorical variables were compared using Chi-squared test.

IBD, inflammatory bowel disease; GI, gastrointestinal; SD, standard deviation; CD, Crohn’s disease; UC, ulcerative colitis.

Groups with differing superscript letters are significantly different at the 0.05 level following pairwise comparisons with Bonferroni post hoc correction.

### 3.1. Nutrient intake

Table 3 shows average daily energy, macronutrient, and micronutrient intakes across the study groups. There were no significant differences in energy or macronutrient [protein, fat, or carbohydrate] intakes across the groups; however, there were significant differences in intakes of numerous micronutrients. Following pairwise comparison, patients with Active IBD had lower intakes of potassium [*p* = 0.008], iron [*p* ,0.001], magnesium [*p* = 0.001], manganese [*p* = 0.012], vitamin C [*p* = 0.001], vitamin K1 [*p* = 0.005], riboflavin [*p* = 0.044], biotin [*p* = 0.001], and folate [*p* = 0.012] compared with healthy controls. Furthermore, patients with Inactive IBD-GI consumed significantly lower intakes of iron [*p* = 0.043], manganese [*p* = 0.012], vitamin C [*p* = 0.001], biotin [*p* = 0.009], and vitamin K1 [*p* = 0.013] than healthy controls. Patients with Inactive IBD consumed significantly less magnesium than healthy controls [*p* = 0.030]. There were no differences in intakes of any other micronutrients between Inactive IBD and healthy controls.

**Table 3.** Energy, macronutrient, and micronutrient intakes across study groups.

	Active IBD [*n* = 65]	Inactive IBD-GI with gut symptoms [*n* = 86]	Inactive IBD without gut symptoms [*n* = 81]	Healthy controls [*n* = 84]	*p*-value
Energy [kcal/d]	1942 [581]	1923 [590]	1990 [494]	2034 [539]	0.601
Protein [g/d]	81 [24]	83 [29]	86 [24]	85 [28]	0.761
Protein [% total E/d]	17 [4]	17 [4]	18 [4]	17 [3]	0.601
Total fat [g/d]	80 [28]	76 [28]	79 [23]	84 [27]	0.245
Fat [% total E/d]	37 [6]	35 [5]	36 [5]	37 [6]	0.140
Saturated fat [g/d]	28 [12]	27 [11]	28 [10]	28 [9]	0.702
Saturated fat [% total E/d]	13 [3]	12 [3]	12 [3]	13.6 [3]	0.687
Monounsaturated fat [g/d]	27 [9]	27 [11]	27 [10]	28 [11]	0.735
Monounsaturated fat [% total E/d]	13 [3]	13 [3]	12 [3]	13 [3]	0.630
Polyunsaturated fat [g/d]	12 [5]	12 [5]	12 [5]	14 [8]	0.144
Polyunsaturated fat [% total E/d]	6 [2]	6 [2]	5 [2]	6 [2]	0.447
Carbohydrate [g/d]	216 [72]	210 [70]	218 [70]	218 [64]	0.875
Carbohydrate [% total E/d]	44 [6]	44 [7]	44 [7]	43 [7]	0.725
Starch [g/d]	130 [41]	126 [43]	124 [34]	124 [42]	0.721
Sugars [g/d]	38 [29]	37 [23]	40 [40]	32 [18]	0.389
Sodium [mg/d]	2145 [712]	2215 [877]	2273 [762]	2189 [671]	0.798
Potassium [mg/d]	2676 [1090]^a^	2886 [1071]^a^^,^^b^	3107 [1273]^a^^,^^b^	3300 [1253]^b^	**0.008**
Calcium [mg/d]	739.9 [297.0]	809.4 [314.0]	835.9 [285.2]	849.2 [291.2]	0.131
Magnesium [mg/d]	259.3 [90.3]^a^	295.0 [137.4]^a^^,^^b^	290.4 [91.0]^a^	344.4 [151.1]^b^	**<0.001**
Phosphorous [mg/d]	1159 [356]	1220 [394]	1268 [338]	1325 [442]	0.061
Iron [mg/d]	9.8 [4.0]^a^	10.9 [3.5]^a^	11.6 [3.8]^a^^,^^b^	12.8 [6.3]^b^	**0.001**
Copper [mg/d]	3.2 [15.9]	20.3 [157.9]	5.4 [27.3]	14.0 [92.1]	0.665
Zinc [mg/d]	8.7 [5.9]	9.2 [6.8]	10.3 [10.0]	10.1 [6.2]	0.509
Chloride [mg/d]	3118 [1051]	3383 [1249]	3390 [1144]	3277 [1004]	0.433
Manganese [mg/d]	3.1 [1.3]^a^	3.8 [3.0]^a^^,^^b^	4.2 [6.5]^a^^,^^b^	5.4 [5.3]^b^	**0.015**
Selenium [μg/d]	52.6 [22.2]	51.1 [19.2]	55.8 [21.7]	57.5 [27.7]	0.256
Iodine [μg/d]	158.0 [385.9]	198.0 [626.3]	120.3 [55.9]	135.5 [81.2]	0.561
Vitamin A [μg/d]	741.4 [530.6]	988.9 [1392.6]	765.3 [437.1]	1105 [898]	0.038
Vitamin E [mg/d]	9.0 [4.2]	9.2 [4.2]	9.7 [4.5]	10.9 [5.4]	0.047
Vitamin D [μg/d]	3.8 [2.7]	3.4 [2.3]	3.5 [2.6]	3.2 [2.5]	0.561
Vitamin C [mg/d]	74.8 [43.1]^a^	84.9 [52.1]^a^	104.7 [66.2]^a^^,^^b^	120.4 [72.1]^b^	**<0.001**
Thiamin [mg/d]	1.3 [0.5]	1.5 [0.5]	1.9 [2.7]	1.7 [0.9]	0.104
Riboflavin [mg/d]	1.4 [0.5]^a^	1.6 [0.6]^a^^,^^b^	1.8 [1.0]^b^	1.7 [0.9]^a^^,^^b^	**0.010**
Niacin [mg/d]	34.5 [13.8]	36.9 [13.8]	37.6 [13.4]	34.8 [12.9]	0.127
Pantothenate [mg/d]	4.9 [1.7]	5.6 [2.3]	5.6 [1.8]	5.6 [2.4]	0.067
Pyridoxine [mg/d]	1.7 [0.6]	1.9 [0.7]	2.1 [1.1]	1.8 [0.7]	0.073
Biotin [μg/d]	32.1 [14.2]^a^	34.9 [13.3]^a^	36.3 [14.5]^a^^,^^b^	43.4 [23.7]^b^	**<0.001**
Folate [μg/d]	196.1 [84.7]^a^	224.8 [99.0]^a^^,^^b^	242.8 [104.1]^a^^,^^b^	248.9 [115.2]^b^	**0.010**
Cobalamin [μg/d]	4.7 [1.9]	5.0 [2.6]	5.2 [2.4]	5.2 [3.8]	0.642
Vitamin K1 [μg/d]	53.7 [57.9]^a^	59.6 [43.3]^a^	77.7 [84.3]^a^^,^^b^	95.3 [99.6]^b^	**0.002**

*p*-values in bold are statistically significant (*p* < 0.05).

Data are mean [SD] daily intake. Groups were compared using ANOVA.

IBD, inflammatory bowel disease; GI, gastrointestinal; SD, standard deviation; d, day; E, energy; ANOVA, analysis of variance.

Groups with differing superscript letters are significantly different at the 0.05 level following pairwise comparisons with Bonferroni post hoc correction.

**Figure 2.**
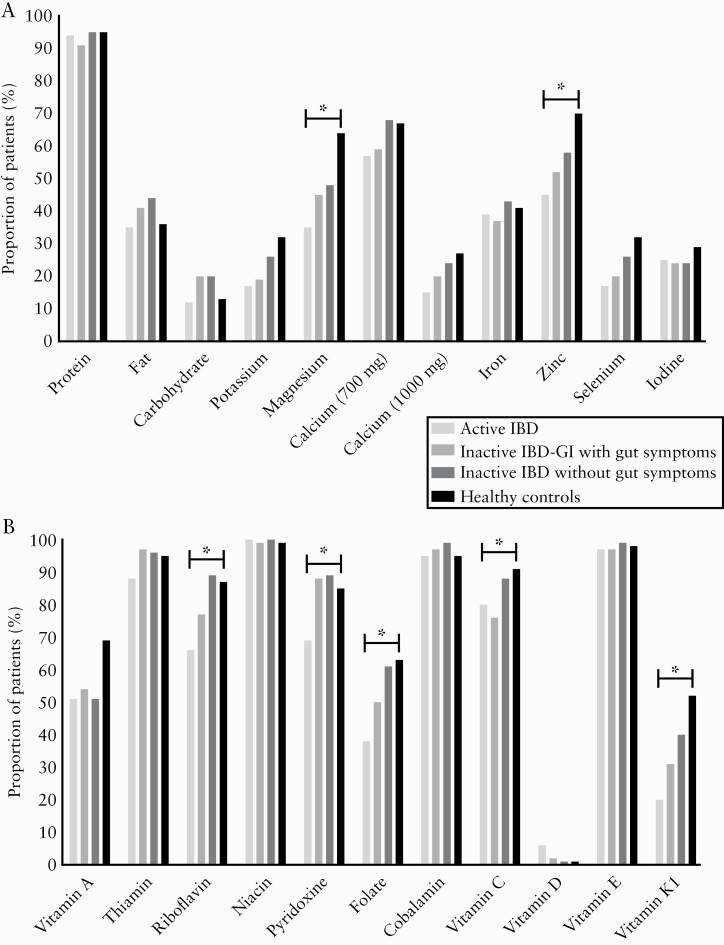
Proportion of participants achieving recommended nutrient intakes across study groups. [A] Proportion of participants achieving recommended macronutrient and mineral intakes. [B] Proportion of participants achieving recommended vitamin intakes. For nutrients marked with an asterisk, the proportion achieving recommendations were significantly different across groups following post-hoc correction.

The proportions of patients achieving dietary reference values for nutrients are shown in Figure 2. Across groups, there were differences in the proportion of patients achieving recommendations for magnesium [*p* = 0.004], zinc [*p* = 0.012], riboflavin [vitamin B_2_] [*p* = 0.002], pyridoxine [vitamin B_6_] [*p* = 0.005], folate [*p* = 0.006], vitamin C [*p* = 0.037], and vitamin K1 [*p* = 0.001]. Compared with healthy controls, significantly fewer patients in the Active IBD group achieved recommended intakes of magnesium [35% vs 64%; *p* = 0.003], zinc [45% vs 70%; *p* = 0.010], vitamin K1 [20% vs 52%; *p* ,0.001], riboflavin [65% vs 87%; *p* = 0.015], and folate [32% vs 63%; *p* = 0.009]. Compared with healthy controls [52%], fewer patients with Inactive IBD-GI achieved recommended vitamin K1 intakes [31%; *p* = 0.033]. The only nutrients with significantly different proportions achieving requirements between the IBD groups were riboflavin, folate, and pyridoxine. Significantly fewer patients with Active IBD achieved pyridoxine recommendations [69%] compared with Inactive IBD-GI [88%; *p* = 0.021] and Inactive IBD [89%; *p* = 0.019]; and compared with Inactive IBD, significantly fewer patients with Active IBD achieved riboflavin [66% vs 89%; *p* = 0.005] and folate [37% vs 60%; *p* = 0.028] recommendations. No significant differences in the proportions of patients achieving vitamin C recommendations were observed between groups upon pairwise comparisons.

### 3.2. Fibre intake

Fibre intakes are presented in Table 4. There was a significant difference in fibre intake across the groups when expressed both as non-starch polysaccharide [NSP] [*p* = 0.001] and total fibre [AOAC method] [*p* = 0.001] as g/d. Pairwise comparisons revealed that compared with healthy controls, there were significantly lower intakes of NSP and total fibre in those with Active IBD [NSP *p* = 0.001, total fibre *p* = 0.001], Inactive IBD-GI [*p* = 0.001, *p* = 0.001] and Inactive IBD [*p* = 0.004, *p* = 0.003], but no differences between the different IBD groups. Mean differences between healthy controls and the IBD groups ranged from 3.2–4.6 g/d for NSP and 4.5–5.8 g/d for total fibre. In order to establish whether lower fibre intakes in IBD reflected a true avoidance of high fibre foods or were simply the result of reduced overall food intake, fibre intakes were calculated per 1000 kcal of energy intake. There was a significant difference in both NSP [*p* = 0.001] and total fibre [*p* = 0.001] intake per 1000 kcal across the study groups. Compared with healthy controls, there were significantly lower NSP and fibre intakes per 1000 kcal in Active IBD [NSP *p* = 0.001, total fibre *p* = 0.001], Inactive IBD-GI [*p* = 0.005, *p* = 0.002], and Inactive IBD [*p* = 0.004, *p* = 0.002], but no differences between IBD groups.

**Table 4.** Intakes of fibre and FODMAPs across the study groups, presented as both g/d and g/1000 kcal/d.

	Active IBD [*n* = 65]	Inactive IBD-GI with gut symptoms [*n* = 86]	Inactive IBD without gut symptoms [*n* = 81]	Healthy controls [*n* = 84]	*p*-value
Non-starch polysaccharide [NSP]					
NSP, g/d, mean [SD]	13.0 [5.0]^a^	13.0 [5.4]^a^	14.4 [5.0]^a^	17.6 [8.1]^b^	**<0.001**
NSP, g/1000 kcal/d, mean [SD]	6.9 [2.3]^a^	7.5 [2.3]^a^	7.3 [3.3] ^a^	8.4 [3.8]^b^	**<0.001**
Total fibre [AOAC]					
Fibre, g/d, mean [SD]	18.9 [7.1]^a^	19.9 [7.4]^a^	20.3 [6.6]^a^	24.7 [11.0]^b^	**<0.001**
Fibre, g/1000 kcal/d, mean [SD]	10.0 [3.3]^a^	10.5 [3.1]^a^	10.4 [3.0]^a^	12.2 [3.8]^b^	**<0.001**
Fibre, *n* [%] achieving RNI [30 g/d]	4 [6.1]^a^	9 [10.5]^a^^,^^b^	6 [7.4]^a^	20 [23.8]^b^	**0.002**
FODMAPs, g/d, median [IQR]					
Total FODMAPs	12.0 [12.5]^a^	13.3 [14.2]^a^^,^^b^	15.1 [10.9]^a^^,^^b^	16.9 [10.5]^b^	**0.003**
Fructans	2.5 [1.6]^a^	2.8 [1.8]^a^^,^^b^	3.1 [1.3]^a^^,^^b^	3.2 [1.6]^b^	**0.008**
GOS	0.9 [0.6]	0.8 [0.8]	1.0 [0.8]	1.0 [1.1]	0.338
Lactose	5.0 [11.3]	7.1 [12.4]	7.1 [10.7]	9.1 [12.3]	0.074
Total fructose	9.6 [7.8]^a^^,^^b^	9.4 [7.6]^a^	11.4 [9.4]^a^^,^^b^	12.6 [8.5]^b^	**0.006**
Excess fructose	0.9 [1.1]	0.8 [1.2]	1.7 [1.3]	1.1 [1.5]	0.215
Sorbitol	0.3 [0.5]^a^	0.4 [0.8]^a^	0.4 [1.0]^a^	0.7 [1.1]^b^	**<0.001**
Mannitol	0.2 [0.4]	0.2 [0.4]	0.2 [0.6]	0.2 [0.7]	0.350
FODMAPs, g/1000 kcal/d, median [IQR]					
Total FODMAPs	5.5 [5.0]^a^	7.4 [7.2]^a^^,^^b^	7.5 [5.6]^a^^,^^b^	8.2 [5.4]^b^	**0.011**
Fructans	1.3 [0.7]^a^	1.5 [0.6]^a^^,^^b^	1.5 [0.5]^a^^,^^b^	1.7 [0.7]^b^	**0.007**
GOS	0.5 [0.3]	0.4 [0.4]	0.5 [0.3]	0.5 [0.5]	0.623
Lactose	2.5 [5.7]	4.8 [6.9]	3.7 [6.2]	4.4 [6.1]	0.059
Fructose	5.3 [3.1]^a^^,^^b^	5.1 [4.1]^a^	5.9 [3.9]^a^^,^^b^	6.7 [4.4]^b^	**0.014**
Excess fructose	0.4 [0.6]	0.4 [0.5]	0.5 [0.7]	0.5 [0.6]	0.335
Sorbitol	0.2 [0.3]^a^	0.2 [0.4]^a^	0.2 [0.5]^a^	0.4 [0.6]^b^	**<0.001**
Mannitol	0.1 [0.2]	0.1 [0.2]	0.1 [0.3]	0.1 [0.3]	0.478

*p*-values in bold are statistically significant (*p* < 0.05).

Continuous data are presented as mean [SD] or median [IQR] and *p*-values represent the result of ANOVA [for normally distributed data] or Kruskal‐Wallis test [for non-normally distributed data] across groups.

IBD, inflammatory bowel disease; GI, gastrointestinal; SD, standard deviation; d,day; AOAC,

Association of Analytical Chemists; RNI, reference nutrient intake; FODMAP, fermentable oligosaccharides, disaccharides, monosaccharides and polyols; GOS, galacto-oligosaccharides; IQR, interquartile range; ANOVA, analysis of variance.

Groups with differing superscript letters are significantly different at the 0.05 level following pairwise comparisons with Bonferroni post hoc correction.

Across groups, there was a significant difference in the proportion of patients achieving recommendations for daily total fibre [AOAC] intake [30 g/d] [*p* = 0.002]. Compared with healthy controls [23.8%], significantly fewer patients achieved total fibre recommendations in the Active IBD [6.1%; *p* = 0.022] and Inactive IBD [7.4%; *p* = 0.023] groups.

### 3.3. Total and individual FODMAP intakes

Total and individual FODMAP intakes are presented in Table 4. When calculated as absolute intakes [g/d], there were significant differences in the intakes of total FODMAPs [*p* = 0.003], fructans [*p* = 0.008], total fructose [*p* = 0.006], and sorbitol [<0.001] across groups. Pairwise comparisons revealed significantly lower total FODMAP [*p* = 0.002] and fructan [*p* = 0.005] intakes in Active IBD compared with healthy controls. Furthermore, there was a lower total fructose intake in Inactive IBD-GI [*p* = 0.008] and lower sorbitol intakes in Active IBD [*p* = 0.001], Inactive IBD-GI [*p* = 0.002], and Inactive IBD [*p* = 0.012] compared with healthy controls. There were no differences in intakes of any FODMAPs between any of the IBD groups. There were no differences in lactose, mannitol, or GOS intakes [g/d] across the groups.

As with fibre intake, it was important to establish whether lower FODMAP intake in IBD reflected a true avoidance of FODMAPs or whether they were simply the result of reduced overall food intake. Across all study groups, there was a significant difference, per 1000 kcal of energy intake, in total FODMAPs [*p* = 0.011], fructans [*p* = 0.007], total fructose [*p* = 0.014], and sorbitol [*p* ,0.001]. Pairwise comparisons revealed that compared with healthy controls, there were significantly lower total FODMAP [*p* = 0.006] and fructan [*p* = 0.003] intakes in Active IBD, lower total fructose intake in Inactive IBD-GI [*p* = 0.019], and lower sorbitol in Active IBD [*p* = 0.001], Inactive IBD-GI [*p* = 0.004], and Inactive IBD [*p* = 0.013]. There were no differences in intakes of any FODMAPs [g/1000 kcal/d] between any of the IBD groups.

### 3.4. Food-related quality of life

**Figure 3.**
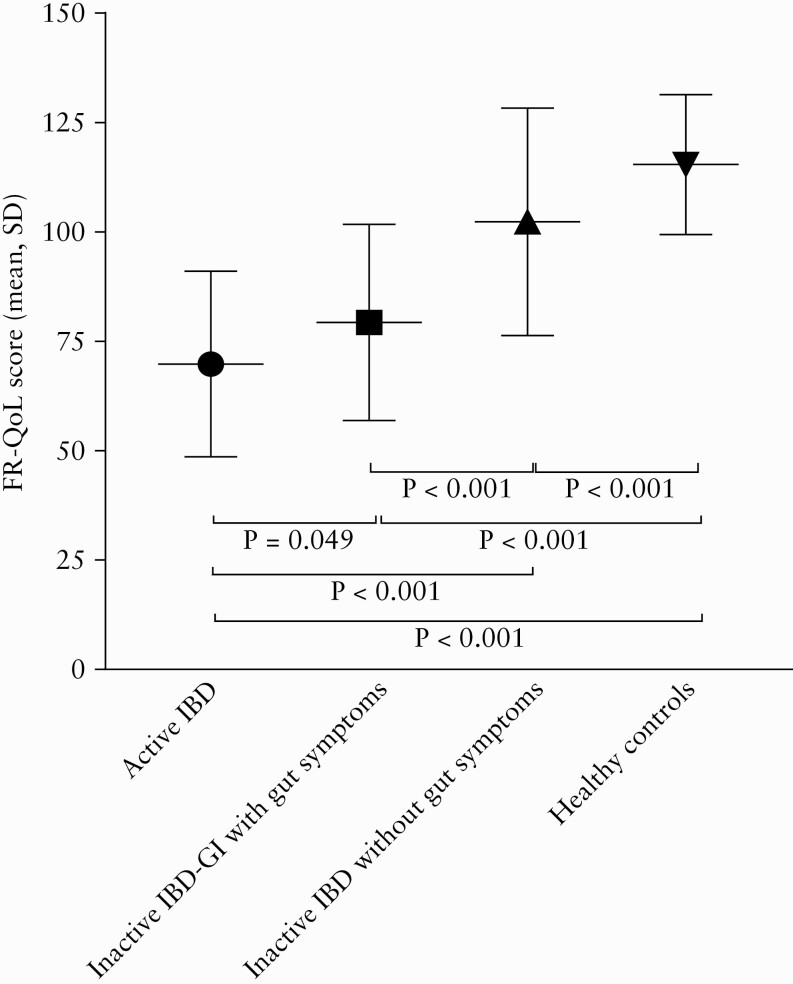
FR-QoL scores across study groups [*p* = 0.001]. Data presented are mean [SD]. FR-QoL, food-related quality of life; SD, standard deviation,

The FR-QoL-29 total score was significantly different across groups [*p* = 0.001] [Figure 3]. Pairwise comparisons revealed significantly different scores for each group compared with all other groups, in a stepwise manner with the lowest score being in patients with Active IBD [mean 69.9, SD 21.2], followed by Inactive IBD-GI [mean 79.3, SD 22.4], Inactive IBD [mean 102.3, SD 26.0], and finally the healthy controls having the highest scores [mean 115.4 SD 16.0].

There was a significant positive correlation between FR-QoL-29 total score and IBD-control score [r = 0.656, *p* = 0.001], and significant negative correlations with total HBI score in CD [r = -602, *p* = 0.001] and total SCCAI in UC [r = -0.440, *p* = 0.001], indicating that greater IBD control and lower clinical disease activity were associated with improved FR-QoL.

## 4. Discussion

This case-control study represents the first robust assessment of dietary intake across multiple groups of patients with IBD, including those with gut symptoms of differing aetiologies [inflammatory GI symptoms in Active IBD, non-inflammatory GI symptoms in Inactive IBD-GI], and patients [Inactive IBD] and healthy controls without gut symptoms. Furthermore, this is the first assessment of FODMAP intakes in patients with IBD with non-inflammatory symptoms compared with other patients with IBD.

There were lower intakes of numerous micronutrients in Active IBD [potassium, iron, magnesium, manganese, vitamin C, vitamin K1, riboflavin, biotin, and folate] and patients with Inactive IBD-GI [iron, manganese, vitamin C, biotin, and vitamin K1] compared with healthy controls. Some of the observed lower nutrient intakes in Active IBD are in line with findings of previous studies.^12,13,17^

Lower intakes of potassium and vitamin C may relate to a restriction of fruits and vegetables, and lower iron intakes in Active IBD may relate to restriction of fortified cereal products, which would also be in line with the lower fibre intakes shown here, or may reflect lower meat intake. The latter would be supported by studies showing that some patients with IBD report meat as a perceived trigger of a flare.^1,17,41^ Fewer patients with Active IBD achieved recommended intakes of magnesium, zinc, vitamin K1, riboflavin, and folate compared with healthy controls, and fewer patients with Inactive IBD-GI achieved recommended vitamin K1 intake. Interestingly, there was no difference in the proportion of patients achieving recommended calcium intakes [either 700 mg or 1000 mg/d] across groups. In the IBD groups, 77–84% of patients failed to achieve the higher 1000 mg/d recommendation set for IBD on the grounds of poorer GI absorption and the likelihood of previous steroid use,^38^ which is in line with a previous study in which >85% of patients with CD failed to consume 1000 mg/d calcium.^16^ Even among the healthy controls, 73% consumed less than 1000 mg/d of calcium, suggesting this target may be unrealistic without supplementation.

This is the first comparison of nutrient intakes in patients with IBD experiencing inflammatory [Active IBD] or non-inflammatory [Inactive IBD-GI] gut symptoms compared with healthy controls, and the findings indicate that gut symptoms of either aetiology have the potential to impair nutrient intakes. Non-inflammatory symptoms in IBD may be considered ‘functional’ or ‘IBS-like’ symptoms. Large proportions of patients with IBS report certain food items to worsen gut symptoms,^42,43^ which could lead to dietary restrictions and result in impaired nutrient intakes.

Whereas the intakes of energy and macronutrients [protein, carbohydrate, and fat] were not significantly different across the study groups, NSP and total fibre [AOAC method] were significantly lower in all IBD groups compared with healthy controls. Similar energy intakes across groups, in addition to differences in NSP and fibre remaining when expressing intakes per 1000 kcal of daily energy intake, suggest specific avoidance of high fibre foods as opposed to merely reflecting a lower overall food intake among the IBD groups. Patients anecdotally avoid visibly ‘fibrous’ foods, such as citrus, celery, and string beans. However, although these foods may be high in fibre, a visibly fibrous appearance does not directly relate to total [AOAC] fibre content, and the absence of visible fibre appearance does not imply absence of total [AOAC] fibre.

This study reports the first evidence of impaired fibre intakes in IBD, with and without inflammatory [Active IBD] and non-inflammatory [Inactive IBD-GI] gut symptoms, compared with healthy controls. To date, studies of nutrient intake in IBD have been small, have included only CD,^13,16^ have lacked a healthy control group,^16–18,44^ or have varied in terms of IBD activity and methods used to measure it.^12,13,16–18^ Given this heterogeneity in study design, drawing parallels with the results of the current study is challenging. Some studies report no differences in fibre intakes between IBD and healthy controls,^12,13^ or between IBD and the national average intake.^18^ One recent cross-sectional study of dietary intake, patterns, and behaviours in 47 patients with IBD observed average fibre intakes of 14.2 g/d for males and 9.7 g/d for females,^44^ although dietary intake was assessed using a food-frequency questionnaire and not prospective food diaries. The larger sample size of the current study may have provided greater power to detect differences in fibre intakes, and furthermore used a more robust dietary assessment [7-day food records].

Lower odds of CD relapse (odds ratio [OR] 0.58) have been observed in the highest compared with the lowest fibre intake group in a cross-sectional analysis.^45^ Therefore, although a randomised controlled trial investigating the effects of low and high fibre intakes on GI inflammation in IBD is lacking, the current finding of significantly lower fibre intakes in all IBD groups, even patients without gut symptoms, is concerning. Many patients perceive high fibre foods to worsen gut symptoms or induce a flare,^1,26,46^ and fibre restriction or a ‘low residue’ diet is frequently advised for symptom or stricture management during IBD flares.^47^ It is not known whether patients continue to follow this advice when in remission, or whether lower fibre intakes reflect advice received from clinicians, self-imposed restrictions due to perceived intolerance, or a combination.

Significant differences in total and individual FODMAP intakes were observed between IBD groups and healthy controls. Significantly lower fructan and total FODMAP intakes in Active IBD compared with healthy controls replicate the findings of a previous case-control study,^23^ and fructan and GOS intakes in the Active IBD group [2.5 g/d, 0.9 g/d] were comparable to those in a previous cross-sectional uncontrolled study of IBD [2.3 g/d, 1.0 g/d].^24^ Despite a relatively small difference in median fructan intake between active IBD and healthy controls [0.7 g/d], there was a wide inter-individual variability demonstrated by the wide difference between the 25% centile in the active IBD group [2.0 g/d] and the 75% centile in the healthy control group [4.1 g/d]. In contrast to the previous case-control study,^23^ intakes of all FODMAPs were analysed in this study. We report, for the first time, lower sorbitol intakes in all IBD groups compared with healthy controls. Neither of the previous studies assessing FODMAP intakes in IBD included an assessment of total energy intake,^23,24^ which was therefore crucial in the current study. Emulating the fibre results, the differences in FODMAP intakes across and between groups remained significant when adjusted for energy intakes, suggesting that lower FODMAP intakes reflected a specific avoidance of foods high in certain FODMAPs, rather than a general reduction in food intake in IBD.

Patients were excluded from this study if they had previously received low FODMAP dietary advice or followed a low FODMAP diet; therefore the lower intakes of FODMAPs in mainly the Active IBD and Inactive IBD-GI groups may reflect self-imposed food restrictions. Some patients with IBD are known to restrict grains and cereals,^1,26,48^ vegetables,^20,26,41,46,49^ and spicy foods [which can contain large quantities of onion and garlic, and therefore fructan],^20,26,41,46,48,49^ all major sources of FODMAPs, during an IBD flare or in an attempt to prevent a flare. This may explain the lower fructan and total FODMAP intakes in Active IBD, and restriction of certain fruits and vegetables, such as stone fruits, avocado and broccoli,^1,20,46,49,50^ may be responsible for the lower sorbitol intakes observed in IBD compared with healthy controls. Polyols [sorbitol, mannitol] exert an osmotic effect in the GI tract^51^ and large doses of sorbitol have laxative effects in healthy volunteers,^52^ such that patients with IBD may elect to avoid high doses of polyols.

Fructans, intakes of which were significantly lower in Active IBD compared with Healthy controls, are prebiotic carbohydrates^53^ that are preferentially fermented by Bifidobacteria.^54^ Certain Bifidobacteria species have immune-regulatory effects^55^ and have been shown in murine models to alleviate chemically induced colitis.^56^*Faecalibacterium prausnitzii*, which generates an anti-inflammatory protein,^57^ may directly ferment fructans or may do so through cross-feeding interactions.^58,59^ Indeed, reduced *F. prausnitzii* abundance was observed following a 4-week low FODMAP diet [restricting fructans] compared with a placebo diet in Inactive IBD.^27^ At surgery for CD, higher abundance of *F. prausnitzii* is associated with lower risk of CD recurrence,^60^ and therefore fructan restriction could have implications in terms of IBD activity, although this has not been established to date.

Interestingly, lactose intakes were not significantly different across study groups, despite evidence that many patients with IBD restrict dairy products or identify them as a symptom trigger.^1,41,61^ Although individuals following special diets [such as a vegan or Paleo diet] were excluded from the healthy control group, some may have been consuming plant-based dairy products, in line with trends in the general population.^62^ This would lower the lactose intake in the healthy controls and thus limit the differences compared with the IBD groups. Despite some studies identifying beans and pulses as a self-reported gut symptom trigger in IBD,^46,48^ GOS intakes were not different across the groups.

FR-QoL encompasses the psychosocial aspects of eating and drinking, such as enjoyment of food and the role of food in social occasions and relationships.^39^ Difficulties around food avoidance, uncertainty around the effects of foods on gut symptoms, and gut symptoms placing restrictions on social occasions involving food [e.g. needing to be close to a toilet], can lead to impaired FR-QoL in IBD.^3^ The current study assesses FR-QoL for the first time in IBD patients with inflammatory [Active IBD] and non-inflammatory [Inactive IBD-GI] gut symptoms compared with IBD patients without gut symptoms [Inactive IBD] and healthy controls. The FR-QoL scores increased in a stepwise fashion from Active IBD to healthy controls and are in line with a previous FR-QoL assessment in IBD and IBS, in which FR-QoL scores increased from the lowest in Active IBD, followed by IBS, and the highest in Inactive IBD.^63^ This indicates that gut symptoms can impair FR-QoL regardless of their aetiology, and furthermore that even patients with IBD without gut symptoms may have impaired FR-QoL.

This study included dietary intake information for in excess of 200 patients with IBD and used 7-day food records, administered by experienced dietitians, making this the largest and most robust assessment of dietary intake in IBD. Furthermore, for the first time, this study objectively differentiated and compared dietary intake between patients with inflammatory symptoms and those with non-inflammatory gut symptoms. An assessment of FODMAP intakes in these distinct IBD groups compared with healthy controls is also novel.

Despite these strengths, there are limitations to the study. Statistical power may have been compromised in the Active IBD group due to a poorer food record return rate, and therefore fewer food records were available compared with the other groups. Despite this, significant differences in nutrient and FODMAP intakes were predominantly observed in Active IBD compared with healthy controls. Significant differences in age and educational level were evident in the IBD groups compared with healthy controls, likely a result of the healthy controls consisting predominantly of university and hospital students and staff. Evidence exists showing that dietary behaviours and intake may be influenced by educational level,^64^ and this should be considered when interpreting the findings of this study. We did not measure attitudes to healthy eating between the groups and so were not able to compare the representativeness of the healthy control population for this domain. Furthermore, in the absence of a FODMAP database of UK foods at the time of data analysis, a database compiled from the analysis of Australian foods^65^ was used to estimate FODMAP intakes. Certain Australian foods may differ in FODMAP content from UK equivalents, and this may have introduced error into the FODMAP analysis. Evidence suggests that dietary exclusions correlate with micronutrient intakes in IBD,^66^ and an assessment of perceived dietary intake and behaviours might have been a useful addition to this study. Finally, this study only measured micronutrient intake and not micronutrient status. This would be an important consideration in future research, although assessing micronutrient status can be challenging in IBD due to the impact of inflammation on some serum markers.

In conclusion, this study has revealed differences in nutrient, fibre, and FODMAP intakes and nutritional adequacy in patients with IBD experiencing gut symptoms of differing aetiologies, compared with patients with Inactive IBD and healthy controls. Notable findings include lower intakes of fructans, sorbitol, and a range of micronutrients among patients with Active IBD compared with healthy controls. Strikingly, lower fibre intakes were observed in all IBD groups compared with healthy controls. Future research should focus on managing these nutritional inadequacies, particularly suboptimal fibre intakes which appear to continue during IBD remission and may adversely affect the gut microbiota.

## Non-Standard Abbreviations

FODMAP: fermentable oligosaccharides disaccharides, monosaccharides and polyols
FFQ: food frequency questionnaire
A-IBD: active IBD
I-IBD-GI: inactive IBD with non-inflammatory symptoms
I-IBD: inactive IBD
HC: healthy controls
FR-QoL: food-related quality of life
NSP: non-starch polysaccharide
AOAC: Association of Analytical Chemists
